# Haemophilia B: an illustrative review of current challenges and opportunities

**DOI:** 10.1016/j.rpth.2025.103229

**Published:** 2025-10-22

**Authors:** Cedric Hermans, Jan Astermark, Sonata Šaulytė Trakymienė, Víctor Jiménez-Yuste

**Affiliations:** 1 Division of Haematology, Cliniques Universitaires Saint-Luc, Brussels, Belgium; 2 Division of Hematology, Université Catholique de Louvain (UCLouvain), Louvain-la-Neuve, Belgium; 3 Department of Translational Medicine, Lund University, Malmö, Sweden; 4 Department of Hematology, Oncology and Radiation Physics, Skåne University Hospital, Malmö, Sweden; 5 Clinic of Children’s Diseases, Institute of Clinical Medicine, Faculty of Medicine, Vilnius University/Vilnius University Hospital Santaros Clinics, Vilnius, Lithuania; 6 Servicio de Hematología, Hospital Universitario La Paz-IdiPaz, Universidad Autónoma, Madrid, Spain

**Keywords:** antibodies, inhibitory, blood coagulation disorders, inherited, factor IX, hemophilia B, hemophilia treatment, hemostatic agents, illustrated review, prophylaxis

## Abstract

**Background:**

Hemophilia B is a genetic bleeding disorder caused by a deficiency of clotting factor IX, which presents unique challenges in clinical management. Advances in therapeutic strategies for hemophilia B have significantly improved patient outcomes but have also necessitated ongoing education for healthcare professionals. This illustrated review provides an overview of the challenges and opportunities in hemophilia B care, including best practice management, emerging therapies, and remaining research needs.

**Objectives:**

To provide a comprehensive visual summary of contemporary perspectives on hemophilia B pathophysiology and management through an illustrated review.

**Methods:**

The authors, leveraging their clinical experience and expertise in hemophilia B management, conducted a review of relevant articles in PubMed (Supplementary Methods).

**Results:**

The review is divided into illustrated sections that provide an overview of hemophilia B, detailing its clinical manifestations, hemostatic agents, and treatment challenges. It also examines the pharmacokinetic properties of hemophilia B and the importance of individualized treatment approaches.

**Conclusion:**

This illustrated review educates healthcare professionals on hemophilia B management in the current treatment landscape, empowering them to further disseminate knowledge to both their colleagues and patients.

## Essentials

• Hemophilia B is a genetic bleeding disorder that presents unique challenges in clinical management.
• This review provides a visual summary of contemporary perspectives on hemophilia B care.
• Nonfactor therapy and gene therapy are novel therapies that will aid treatment individualization.
• Healthcare professionals and patients require ongoing education on the evolving treatment landscape.

**Figure:**
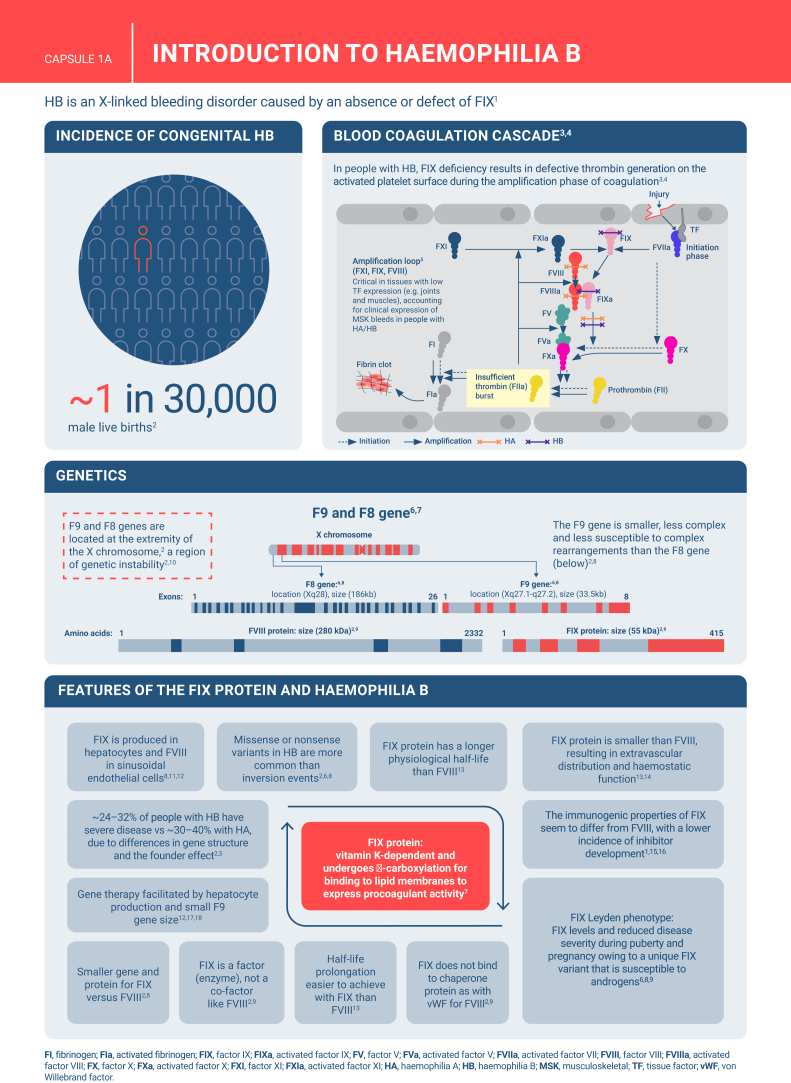

**Figure:**
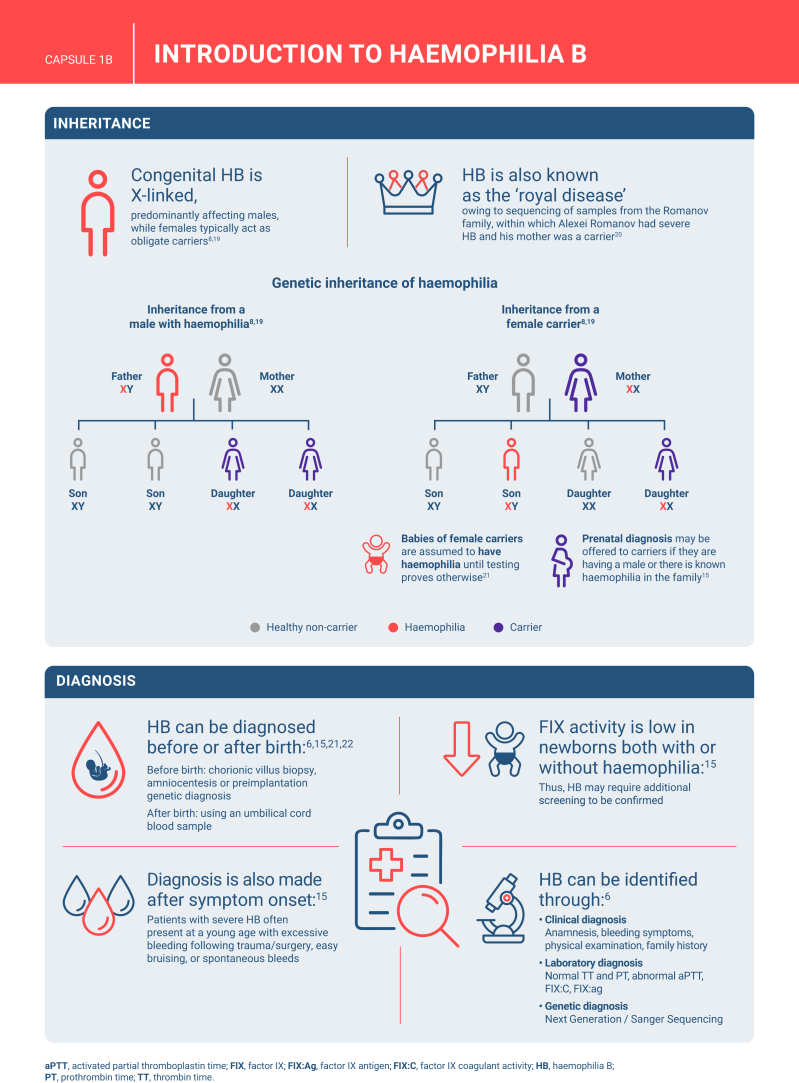

**Figure:**
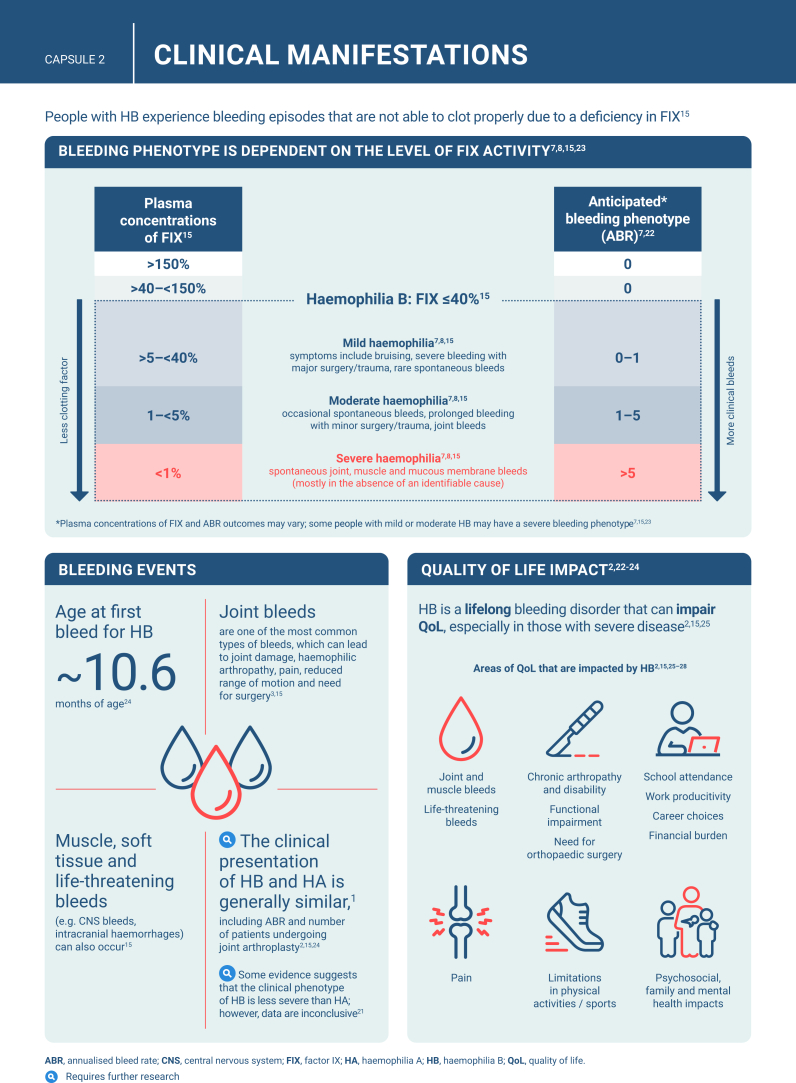

**Figure:**
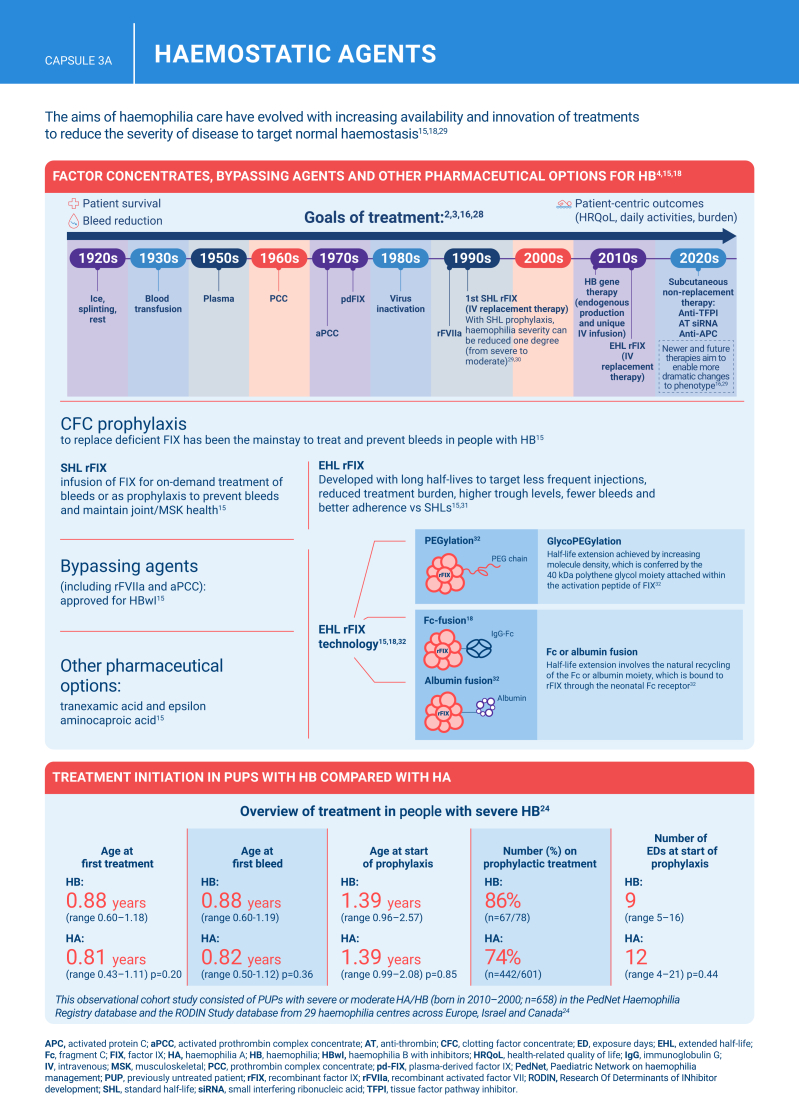

**Figure:**
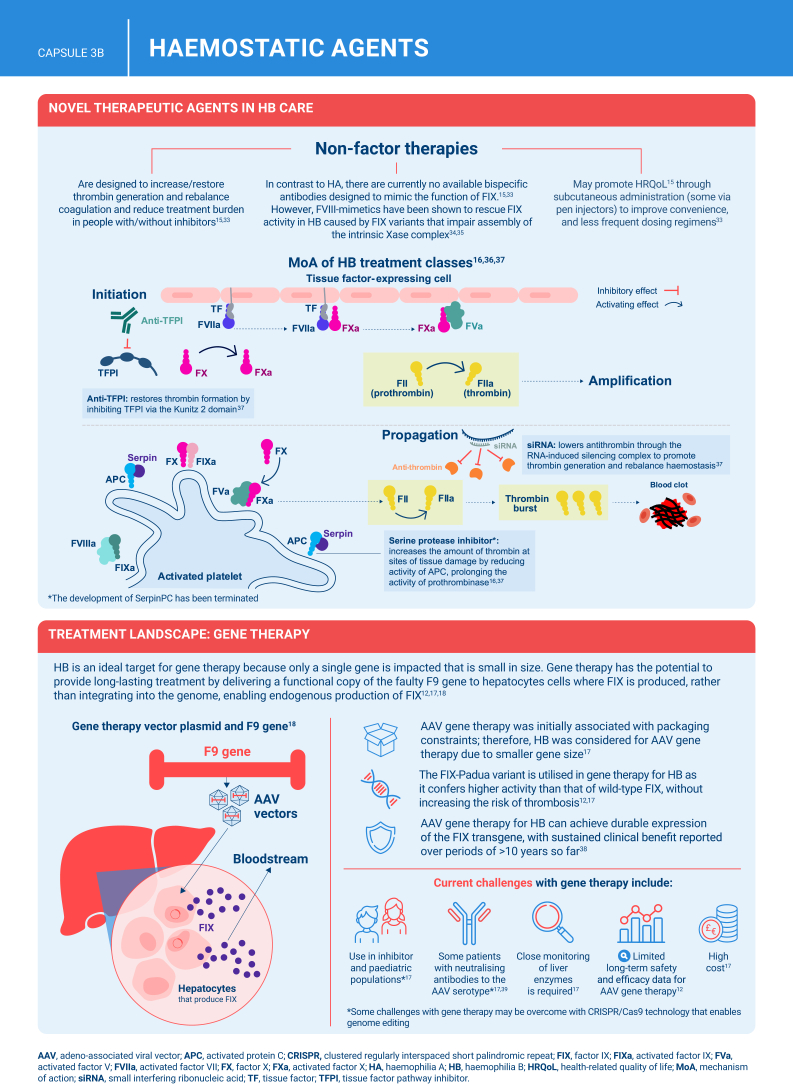

**Figure:**
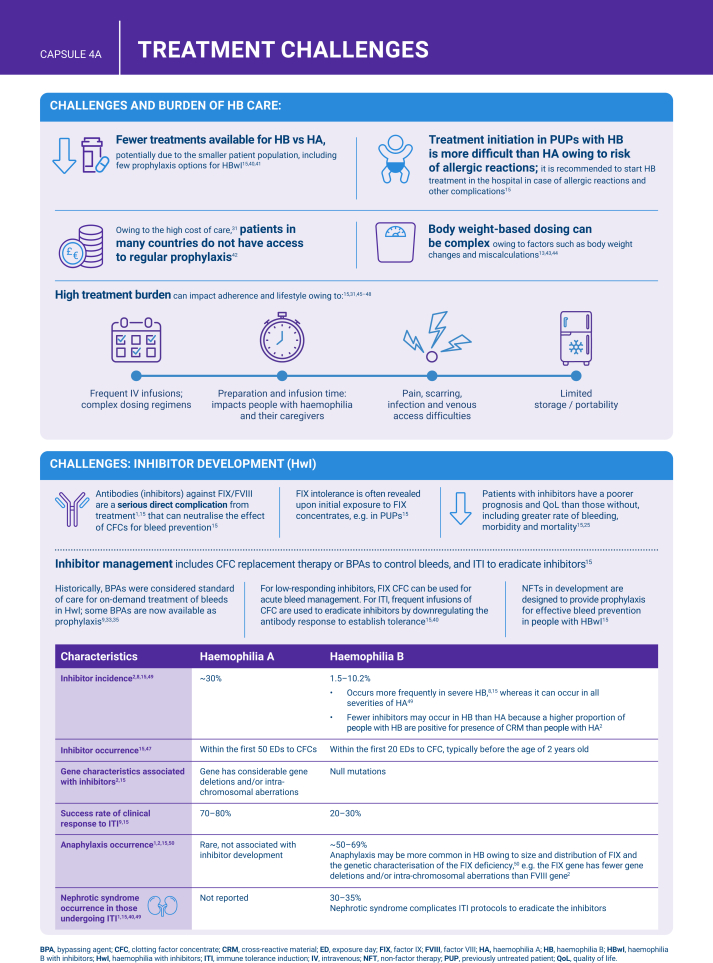

**Figure:**
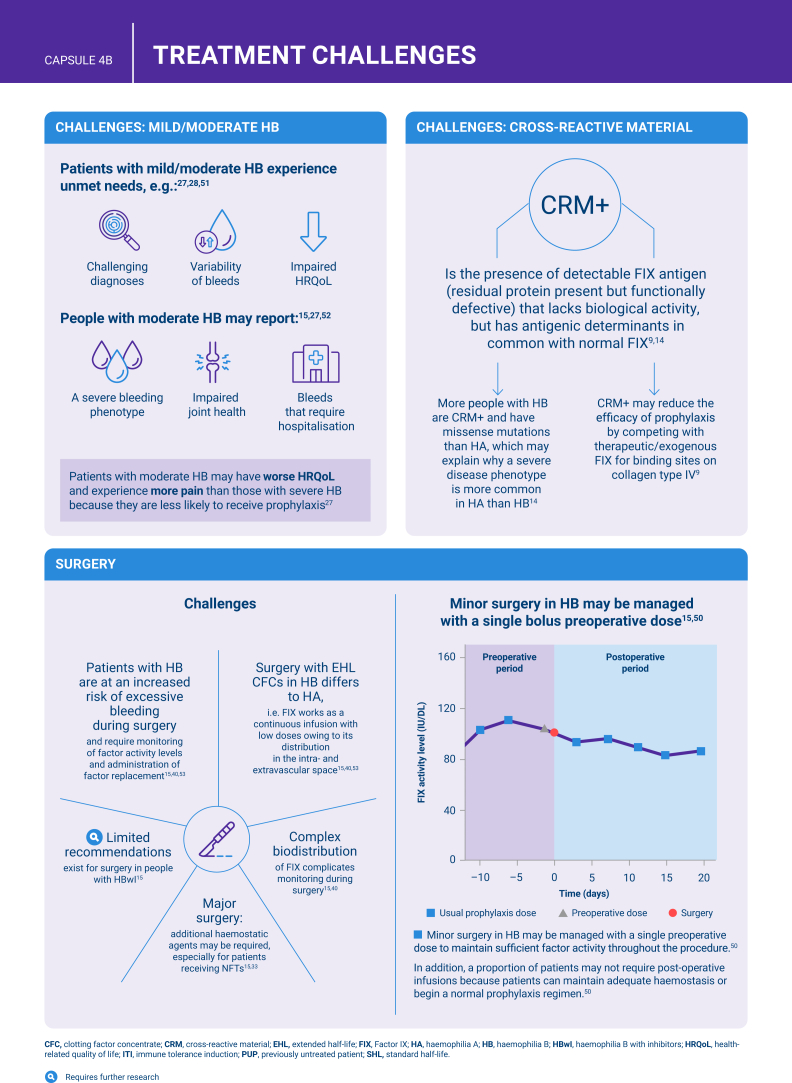

**Figure:**
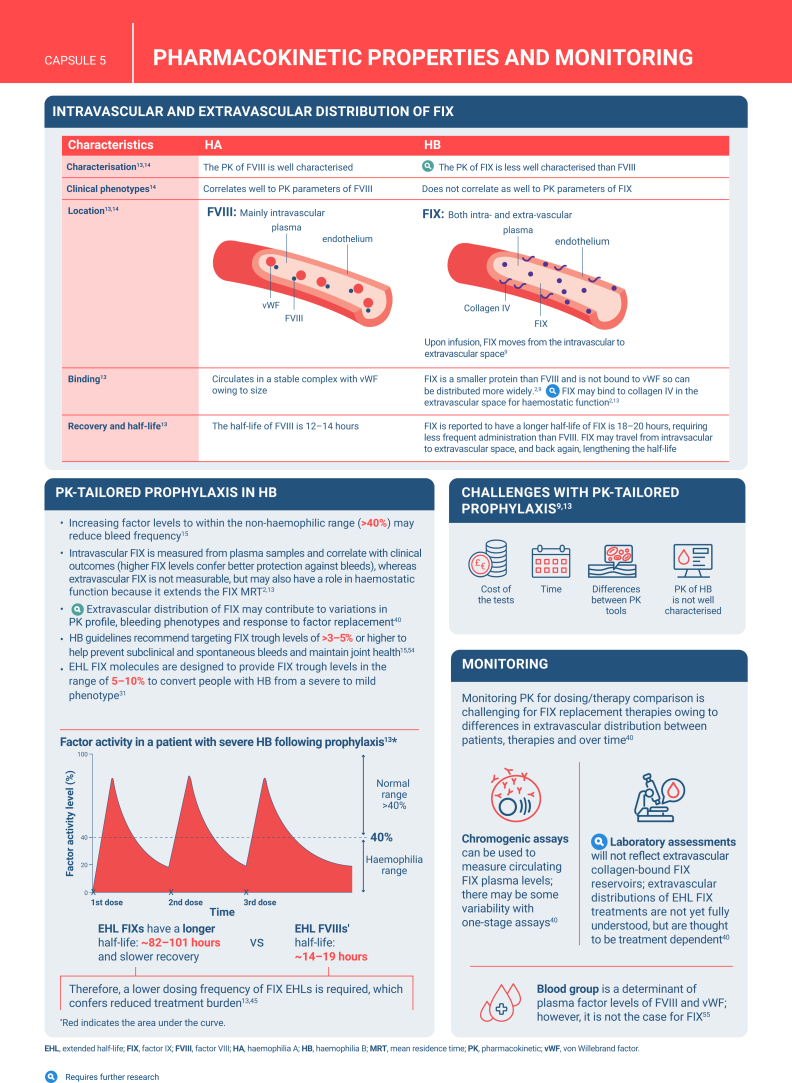

**Figure:**
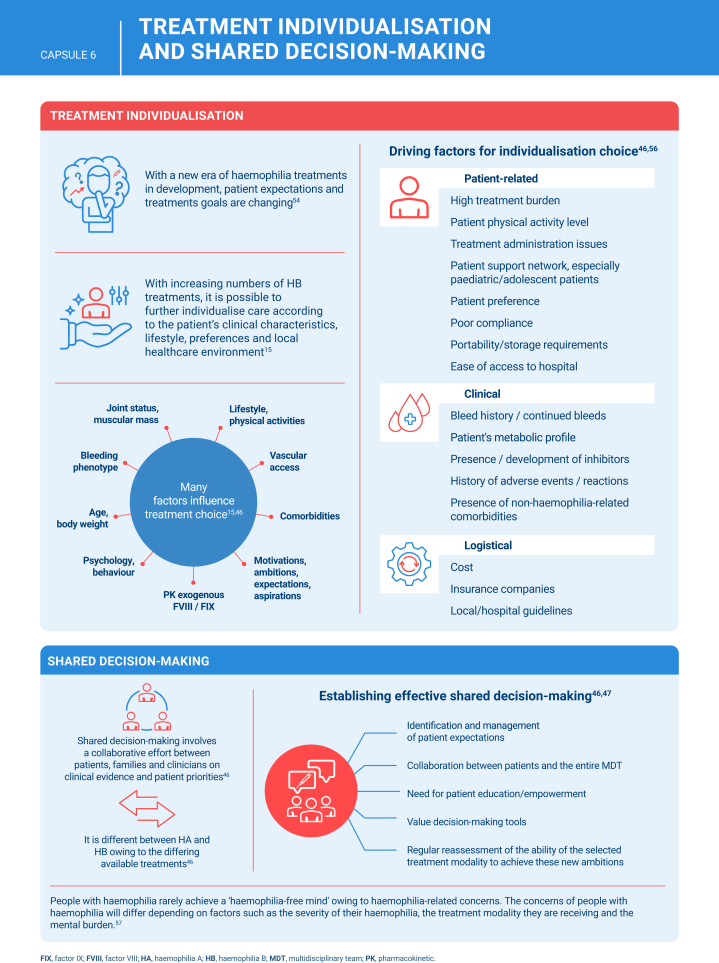

**Figure:**
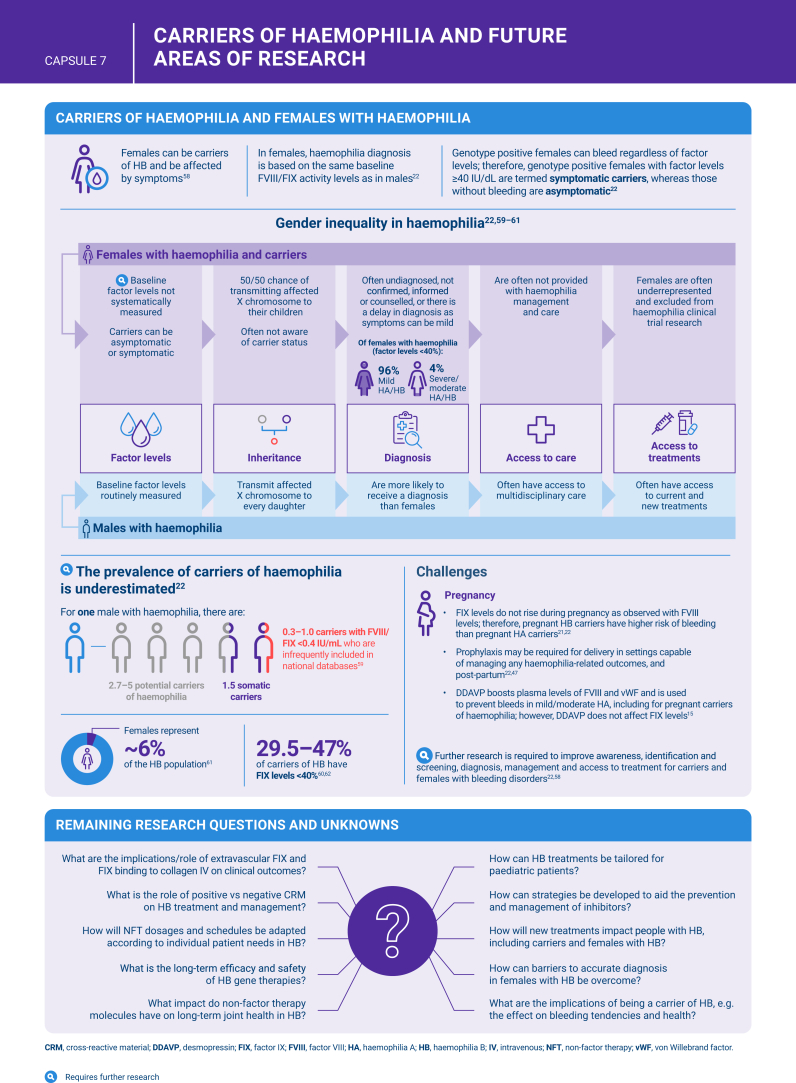

## Supplementary material

Illustrative Review Supplementary
