# Supplementary material for: Haemophilia B: an illustrative review of current challenges and opportunities
Source: Res Pract Thromb Haemost. 2025 Oct 22;9(8):103229. doi: 10.1016/j.rpth.2025.103229 (PMC12663517; doi:10.1016/j.rpth.2025.103229)
Supplement: Illustrative Review Supplementary [file mmc1.docx]

**Supplemental Methods**

**Manuscript title:**

Haemophilia B: An illustrative review of current challenges and opportunities

**Further methodology information:**

An initial literature search was conducted on PubMed and included (but was not limited to) the following search terms: (“haemophilia B” OR “factor IX deficiency”) AND (“clinical guidance” OR “guideline” OR “management” OR “treatment” OR “unmet need” OR “gene therapy” OR “non-factor therapy” OR “novel therapy” OR “inhibitors” OR “symptoms” OR “manifestations” OR “treatment challenges”). Additional searches of PubMed for peer-reviewed articles were performed to provide more granular detail on key research topics included. No formal time restrictions for article selection were applied, but articles from 2018 onwards were prioritised to reflect contemporary perspectives of haemophilia B management. The authors leveraged their clinical experience and expertise in haemophilia B management to objectively assess the quality of discussion points and evidence included.
